# Flaxseed (*Linum Usitatissimum* L.) Supplementation in Patients Undergoing Lipoprotein Apheresis for Severe Hyperlipidemia—A Pilot Study

**DOI:** 10.3390/nu12041137

**Published:** 2020-04-18

**Authors:** Dominika Kanikowska, Katarzyna Korybalska, Agnieszka Mickiewicz, Rafał Rutkowski, Agnieszka Kuchta, Maki Sato, Ewelina Kreft, Marcin Fijałkowski, Marcin Gruchała, Maciej Jankowski, Andrzej Bręborowicz, Janusz Witowski

**Affiliations:** 1 Department of Pathophysiology, Poznan University of Medical Sciences, Rokietnicka 8, 60-806 Poznań, Poland; koryb@ump.edu.pl (K.K.); rrutkowski@ump.edu.pl (R.R.); abreb@ump.edu.pl (A.B.); jwitow@ump.edu.pl (J.W.); 2 1st Department of Cardiology, Medical University of Gdansk, Marii Skłodowskiej-Curie 3a, 80-210 Gdańsk, Poland; agnieszka.mickiewicz@gumed.edu.pl (A.M.); marcin.fijalkowski@gumed.edu.pl (M.F.); marcin.gruchala@gumed.edu.pl (M.G.); 3 Department of Clinical Chemistry, Medical University of Gdansk, Marii Skłodowskiej-Curie 3a, 80-210 Gdańsk, Poland; agnieszka.kuchta@gumed.edu.pl (A.K.); ewelina.kreft@gumed.edu.pl (E.K.); maciej.jankowski@gumed.edu.pl (M.J.); 4 Department of Physiology, School of Medicine, Institutional Research, Aichi Medical University, Nagakute, Aichi 480-1195, Japan; msato@aichi-med-u.ac.jp

**Keywords:** flaxseed, lipoprotein apheresis

## Abstract

Being rich in polyunsaturated fatty acids, flaxseed (*Linum usitatissimum* L.) is thought to be able to decrease lipid levels and dampen inflammation. In this pilot study, we aimed to determine whether flaxseed supplementation could improve the profiles of lipids and inflammatory mediators in patients with severe hyperlipidemia resistant to conventional lipid-lowering pharmacotherapy and requiring lipoprotein apheresis. To this end, six patients received, blindly—in addition to their normal lipoprotein apheresis regimen—a 10-week dietary supplementation with flaxseed (28 g/d) administered in biscuits. This was followed by a 10-week washed out-period and a 10-week supplementation phase with whole wheat placebo. Blood samples were collected at the end of each phase, before the lipoprotein apheresis session. The primary endpoint was the lipid profile and the secondary endpoints were the concentrations of inflammatory mediators and tolerability. Flaxseed supplementation was well-tolerated and resulted in a consistent and significant decrease in total cholesterol and low-density lipoprotein (LDL) levels. The median (and range) percentage decrease was 11.5% (0–18.8) and 7.3% (4.4–26.6), for cholesterol (*p* = 0.015) and LDL-C (*p* = 0.003), respectively. On the other hand, there was no significant effect of flaxseed on lipoprotein(a) (Lp(a)), C-reactive protein (CRP), and interleukin 6 (IL-6) concentrations. These observations indicate that flaxseed can produce a cholesterol- and LDL-lowering effect in patients treated with lipoprotein apheresis. Thus, flaxseed supplementation may help to control cholesterol in this patient population. The flaxseed supplementation protocol applied may be of use for further adequately-powered studies to validate and extend our findings.

## 1. Introduction

Adverse effects related to high blood cholesterol are well recognized and include atherosclerosis and cardiovascular disease [1,2]. In particular, high low-density lipoprotein (LDL) cholesterol is considered to confer an increased risk of atherosclerosis progression and cardiovascular events [3]. Additionally, elevated lipoprotein(a) (Lp(a)) is a known risk factor for cardiovascular disease [4,5]. Thus hyperlipidemia-targeting strategies are paramount to disease prevention and include both pharmacotherapy and lipoprotein apheresis. These, however, still have some limitations related to both potential side effects and patient adherence [6,7].

Nonpharmacological management of severe hypercholesterolemia is challenging and includes lifestyle changes. These may be more appealing to patients while reducing the risk of cardiovascular disease. Traditional food products, including vegetable oils, contain components such as *n*-6 and *n*-3 polyunsaturated fatty acids (PUFAs) or monounsaturated fatty acids (MUFAs), which display anti-inflammatory and lipid-lowering activities that are important for the prevention of inflammatory [8,9] and cardiovascular diseases [10,11,12].

Flax (*Linum usitatissimum* L.) is rich in PUFAs, fibre and phytosterols, which contribute to its lipid-lowering and antiatherogonic activity. Of PUFAs, flax contains 15.82% and 56.93% of linoleic acid and α-linolenic acid (ALA), respectively [13]. In particular, ALA and its metabolites are thought to exert beneficial cardiovascular effects [10]. Flaxseed is also the most abundant source of antioxidant lignans and phytosterols. Phytosterols can reduce blood LDL by decreasing cholesterol absorption [14], and displaying anti-inflammatory and antioxidative properties [15,16]. It has been estimated that consumption of 1.5–2.0 g per day of plant sterols reduces the concentration of plasma LDL by 9–14% [17].

Therefore, flaxseed can be recommended as a functional food for patients with hypercholesterolemia. The additional advantage is that flax seeds can be stored, processed, and incorporated easily into many food products.

While the favorable properties of flaxseed are recognized, it is not clear whether patients with severe hypercholesterolemia can benefit from flaxseed supplementation. These are often patients with familial hypercholesterolemia and/or elevated Lp(a), who require regular lipoprotein apheresis to reduce plasma lipids. Thus, in this pilot study, we wished to determine whether flaxseed supplementation can help to control blood cholesterol and reduce the inflammatory burden in patients requiring lipoprotein apheresis.

## 2. Methods

### 2.1. Study Design

**Figure 1:**
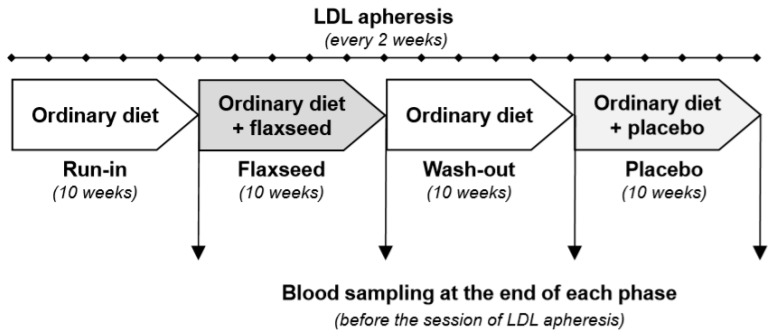
A protocol of the study. LDL, low-density lipoprotein.

This was a pilot, single-centre, prospective study with a single arm and with the phases of treatment and placebo in a predetermined single sequence. All patients underwent an initial 10-week run-in phase on an ordinary diet, after which they received flaxseed supplementation for 10 weeks. This was followed by a wash-out period of 10 weeks on an ordinary diet. After that patients received supplementation with a placebo for a further 10 weeks (Figure 1). The patients were blinded as to what type of supplementation they received. The primary endpoint was the concentration of total cholesterol, LDL-C, and Lp(a). Secondary endpoints were (1) the concentrations of CRP, tumor necrosis factor alpha (TNFα), IL-6, soluble interleukin 6 receptor (sIL-6R), and soluble gp130 (sgp130), and (2) tolerability. 

### 2.2. Study Population

A total of 8 patients receiving lipoprotein apheresis (once every two weeks) for severe hyperlipidaemia were registered. They were undergoing lipoprotein apheresis in the 1st Department of Cardiology of the Medical University of Gdansk. Indications for lipoprotein apheresis were as follows:

1. Statin intolerance and severe hypercholesterolemia with LDL-C > 130 mg/dL, despite treatment for >12 months with maximally tolerated doses and documented atherosclerosis;
2. Severe familial hypercholesterolemia with LDL-C > 130 mg/dL despite treatment for >12 months with maximally tolerated doses, documented atherosclerosis and coexisting elevated Lp(a) >60 mg/dl;
3. Severe hyperlipoproteinemia (a) with Lp(a) level >100 mg/dL in patients with documented atherosclerosis.

Familial hypercholesterolemia was diagnosed using the modified Dutch Lipid Clinic Network Score, validated for the Polish population [18]. Statin intolerance was defined as the inability to tolerate at least two statins: one at the lowest starting daily dose and the other at any daily dose. All patients gave written informed consent and entered the study. An a priori power analysis indicated that a minimum of 8 participants would be required to achieve a power of 0.80 with an α for the ANOVA set at 0.05, and with a moderate-to-large effect size (f2 = 0.50–0.80). However, two patients discontinued treatment and did not complete the study because of relocation (*n* = 1) and additional disease (retinal detachment, *n* = 1). For the planned endpoints, the data from 6 preprotocol patients were analyzed. Patient baseline characteristics are given in Table 1. The study was approved by the Ethics Committee of the Poznan University of Medical Sciences (No. 333/15) and followed the principles of the Declaration of Helsinki. Detailed individual patient characteristics, including lipids before lipoprotein apheresis initiation, cardiovascular (CV) diseases, CV risk factors, and lipid-lowering medications are presented in Table S1.

### 2.3. Flaxseed Supplementation and Study Protocol

Flaxseed was administered for 10 weeks at a dose of 28 g/d, based on the reports by Dittrich et al. (2014) [19], and Pan et al. (2009) [20]. Ground flaxseed was given in biscuits, based on the concept of Wong et al. (2013) [21]. Biscuits were prepared and baked especially for the project (Table 2). Biscuits used as a placebo contained the same amount of whole wheat flour instead of flaxseed. Patients were advised by a qualified dietician before each phase of the study and consumed the required number of biscuits at the same time of day (midmorning).

All nutritional interventions were carried out in addition to regular lipoprotein apheresis treatment and lipid-lowering medication, which did not change during the study period. Participants had blood sampled on 4 occasions (Figure 1). Routine biochemical tests were performed by the same university hospital laboratory. 

Serum samples for cytokine measurements were aliquoted and stored at −80 °C until assayed in batch in the same laboratory. Concentrations of IL-6 and CRP were determined by immunoassays from BioVendor (Brno, Czechia) and concentrations of TNFα, sIL-6R, and sgp130 were measured by immunoassays from R&D Systems Inc. (BioTechne, Minneapolis, MN, USA). All tests were performed as per manufacturers’ instructions. The detection limits were as follows: IL-6—0.32 pg/mL, TNF-alpha—0.049 pg/mL, sgp130—0.05 ng/mL, sIL-6R—15.1 pg/mL, and CRP—0.02 µg/mL. 

### 2.4. Statistical Analysis

All statistical analyses were performed using GraphPad Prism 8.0 (GraphPad Software, La Jolla, CA, USA). Normality of the data distribution was tested with the Shapiro–Wilk’s test. Since the data did not follow a Gaussian distribution, they were analyzed with the nonparametric Friedman test with the Dunn post hoc test for comparisons between the treatments with flaxseed and placebo. The results are presented as individual data with the medians and interquartile ranges indicated. A value of *p* < 0.05 was considered significant. 

## 3. Results

### 3.1. Effect of Flaxseed on Total Cholesterol, LDL and Lp(a) Levels

**Figure 2:**
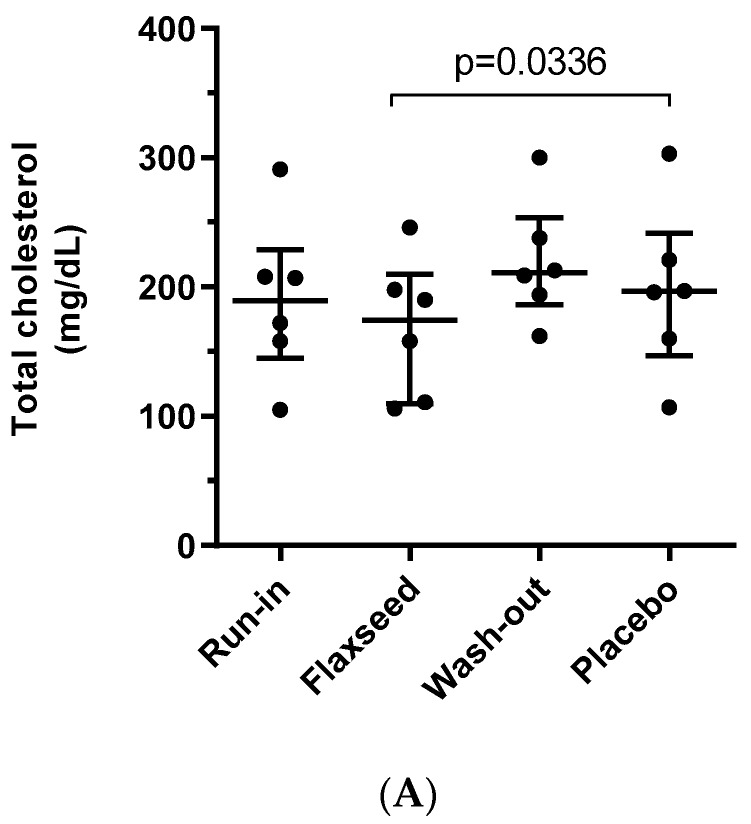

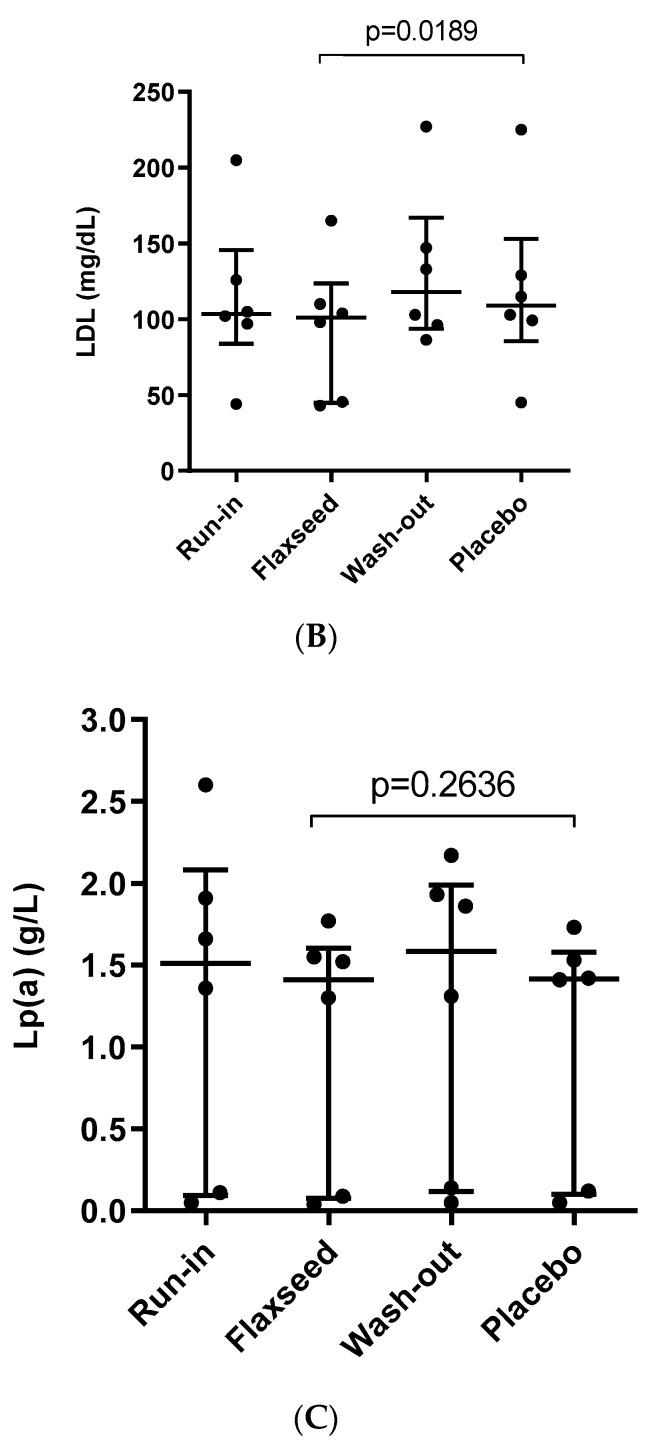
Changes in plasma lipids throughout the study. The concentration of (**A**) total cholesterol; (**B**) LDL; (**C**) Lp(a). The data are given as individual data with medians and ranges indicated, *n* = 6.

Total cholesterol levels were significantly lower after treatment with flaxseed compared with placebo (Figure 2A). This effect was seen in all patients and the median cholesterol level was lower by 11.5% (range 0–18.8). Likewise, LDL levels after treatment with flaxseed were lower in all patients compared with the treatment with placebo (Figure 2B). The difference between medians was 7.3% (range 4.4–26.6). In contrast, there was no significant difference between the treatments in Lp(a) levels (Figure 2C). Interestingly, there appeared to be two clusters of patients differing markedly in baseline Lp(a) levels. Flaxseed supplementation did not alter Lp(a) in either group. Detailed patient’s changes in raw lipids parameters throughout the study, are presented in Table S3.

### 3.2. Effect of Flaxseed on Parameters of Inflammation

There was no significant effect of flaxseed supplementation on CRP, TNFα, IL-6, and sgp130 levels (Table 3). Levels of sIL-6R after treatment with flaxseed were marginally lower (*p* = 0.057) compared to placebo. 

### 3.3. Other Parameters

Compared with placebo, levels of HDL, triglycerides, apoA1, and apoB did not change significantly following flaxseed supplementation (Table 4). 

### 3.4. Detailed Lipid Parameters before and after Lipoprotein Apheresis Sessions

Detailed lipid parameters before and after lipoprotein apheresis sessions are summarized in supplemental data in Table S2. Median preapheresis values of LDL and Lp(a) were high (111 (IQR 51) (91–142) mg/dL and 1.44 (IQR 1.72)(0.11–1.82) g/L, respectively). Apheresis reduced both lipids acutely to 31 (IQR 27)(22–49) mg/dL and 0.3 (IQR 0.43)(0.03–0.46) g/L, respectively. The reductions of LDL up to 71% (IQR 10) (66–76) mg/dL and Lp(a) up to 70% (IQR 16.08) (60–76) g/L were achieved.

### 3.5. Side Effects and Tolerability

Both flaxseed and placebo biscuits were readily consumed and well-tolerated. No gastrointestinal or other adverse effects were reported.

## 4. Discussion

The main observation of the present study is that flaxseed supplementation produced consistent cholesterol- and LDL-lowering effect in patients undergoing regular lipoprotein apheresis. Given the severity of hypercholesterolemia in these patients, the effect of flaxseed can be viewed as very promising and justifying further studies. There have been no earlier reports indicating that flaxseed can be of benefit for such a patient population. A recent meta-analysis of sixty-two randomized controlled trials with a total of 3772 participants suggested that flaxseed supplementation can reduce total serum cholesterol, triglyceride, and LDL in unhealthy subjects with high baseline lipids level [22]. Our pilot study confirms and extends these observations. It shows that flaxseed supplementation for 10 weeks is feasible and well-tolerated and is capable of reducing total cholesterol and LDL by 5–10% even in patients for whom lipoprotein apheresis seemed to be the only treatment option available. As these patients also received pharmacotherapy, the effect of flaxseed is even more impressive. 

Flaxseed demonstrates strong antiatherogenic activity [23], which is thought to be partly related to high contents of ALA [24] with anti-inflammatory and anti-proliferative properties [25]. ALA is metabolized to eicosapentaenoic and docosahexaenoic acids, which activate peroxisome proliferator-activated receptors that control the expression of several key genes involved in lipid metabolism, glucose homeostasis, and adipogenesis [26].

In contrast to LDL and cholesterol, we did not observe a significant reduction in Lp(a) levels following flaxseed treatment. This is in line with earlier observations [22,27], which also did not demonstrate a consistent effect of flaxseed on Lp(a). This lack of a significant effect could be linked to the severity of disease in our patients and their persistently high lipid levels requiring apheresis. It has been reported [28] that the efficiency of cholesterol-lowering therapies is highly related to baseline lipid levels. A randomized, clinical trial in children aged 8 to 18 years with moderate to severe dyslipidemia (*n* = 32) with (30 g/d) flaxseed supplementation found no effect at four weeks on levels of TC or LDL, but an increase in HDL-cholesterol [21]. Different effects observed in our study could be related to a different patient population (adults with higher basal lipid concentrations) and longer treatments period (4 vs. 10 weeks). Other research has also pointed at the possible involvement of genetic background in mechanisms underlying patients’ responses to plant sterols and stanols [29].

Interestingly, we did not observe a reduction in HDL, triglycerides, and other lipids after flaxseed supplementation. This may indicate that flaxseed is targeting the cholesterol pathway more specifically. The lack of apparent effect of flaxseed on triglycerides may also be associated with a wide range of concentrations seen in our small group of patients. We also did not record any significant effect of flaxseed on biochemical parameters of inflammation, which is in contrast to some studies showing a significant reduction in CRP and IL-6 levels [30,31]. On the other hand, other reports also did not detect significant changes in IL-6 and TNFα in response to PUFA-rich diets [32]. The absence of significant changes in our patients could be related to the fact that levels of inflammatory parameters (as exemplified by CRP) were already very low at baseline. Interestingly, we still observed a little reduction in serum sIL-6R. This could potentially impact on the process of IL-6 trans-signaling, which has recently been associated with an increased cardiovascular risk [33]. Circulating sIL-6R is thought to be primarily derived from ectodomain shedding of the membrane-bound IL-6R mediated by metalloprotease ADAM17 [34]. In this respect, Speck et al., (2015) [35] showed that dietary free fatty acids can directly modulate ADAM17 activity in mice. 

## 5. Conclusions

Being a pilot hypothesis-driven exploration, our small-scale study has some obvious limitations. On the other hand, it clearly demonstrates that the applied protocol of flaxseed supplementation gains patients’ acceptance and is well-tolerated. Moreover, it demonstrates a significant cholesterol-lowering effect in such complex patients as those treated with lipoprotein apheresis. This may be of clinical relevance and indicate that flaxseed should be included in the diet regularly. In our opinion, these observations warrant further studies in much larger patient populations, possibly stratified for such factors as the nature of hypercholesterolemia and baseline LDL concentration, age, gender, genetic background, and medical center.

## Figures and Tables

**Table 1:** Baseline characteristics of the participants. The data are given as medians and range, *n* = 6.

Sex, M/F	4/2
Age, years	55.5 (48.0–79.0)
BMI, kg/m^2^	29.5 (22.0–32.0)
Underlying condition:	
Isolated hyper-Lp(a)	2
Familial hypercholesterolemia (confirmed)	1
Clinical phenotype of familial hypercholesterolemia	3
Statin intolerance	2
Ever smoked, Y/N	3/3
Coronary artery disease, Y/N	5/1
Stroke, Y/N	0/6
Diabetes, Y/N	0/6
Hypertension, Y/N	3/3
Time on lipoprotein apheresis, months	26.5 (19.0–38.0)
Lipid profile before commencement of lipoprotein apheresis:	
Lp(a), g/L Reference range	1.1 (0.06–3.1) 0.02–0.74
Total cholesterol, mg/dL Reference range:	239.5 (191.0–367.0) 115–190
LDL, mg/dL Reference range:	152.8 (118.0–152.5) <100
HDL, mg/dL Reference range:	49.0 (39.0–77.0) F > 45; M > 40
Triglycerides, mg/dL Reference range:	294.5 (187.0–735.0) <150
ApoA1, g/L Reference range:	1.6 (1.3–1.8) 1.25–2.15
ApoB, g/L Reference range:	1.1 (0.7–2.0) 0.55–1.25
ApoB/ApoA1 Reference range:	0.7 (0.4–1.2) 0.3–0.9
Additional medication:	
Statins, Y/N	4/2
Ezetimibe, Y/N	5/1
Fenofibrate, Y/N	1/5

Abbreviations: Y, yes; N, no; F, female; M, male; BMI, body mass index; Lp(a), lipoprotein(a); HDL, high density lipoprotein; LDL, low density lipoprotein; ApoA1, apolipoprotein A1; ApoB, apolipoprotein B.

**Table 2:** Nutritional value of experimental biscuits per daily dose. Composition of experimental biscuits (expressed as per daily dose). Abbreviations: FA—fatty acids.

Composition	Flaxseed Biscuit	Placebo Biscuit
Energy, kcal	274.2	230.0
Protein, g	7.0	3.0
Carbohydrates, g	19.6	30.4
Total fat, g	19.2	11.0
• Saturated FA, g	7.0	7.0
• Monounsaturated FA, g	4.2	3.0
• Polyunsaturated FA, g	6.0	0.4
• Cholesterol, mg	33.2	33.2
• Omega-6 FA, g	1.4	0.4
• Omega-3 FA, g	4.8	0.08
Fibers, g	1.0	0.6

**Table 3:** Changes in inflammatory parameters throughout the study. The data are given as medians and ranges, *n* = 6.

Parameter	After				*p*-Value Flaxseed vs. Placebo
	Run-in	Flaxseed	Wash-out	Placebo	
IL 6 (pg/mL)	11.0 (7.7–37.0)	12.7 (8.3–19.3)	11.4 (7.5–35.7)	10.2 (7.7–55.0)	*p* = 0.371
sIL-6R (pg/mL)	53.1 (45.0–75.1)	51.3 (41.3–80.3)	51.9 (38.7–93.0)	65.9 (42.7–81.0)	*p* = 0.057
CRP (µg/mL)	0.46 (0.30–2.93)	0.41 (0.28–0.88)	0.54 (0.34–1.50)	0.53 (0.32–1.10)	*p* = 0.179
TNFα (pg/mL)	0.56 (0.39–0.73)	0.57 (0.31–1.00)	0.40 (0.35–1.05)	0.54 (0.28–1.04)	*p* = 0.654
sgp130 (ng/mL)	490.0(326.8–1287.0)	563.0 (304.0–1920.0)	509.2 (434.5–1067.0)	507.9 (445.9–579.7)	*p* = 0.179

Abbreviations: IL-6, interleukin 6; sIL-6R, soluble interleukin 6 receptor; CRP, C reactive protein; TNF alpha, tumor necrosis factor-alpha; sgp130, soluble gp130.

**Table 4:** Changes in lipids parameters throughout the study. The data are given as medians and range, *n* = 6.

Parameter	After				*p*-Value Flaxseed vs. Placebo
	Run-in	Flaxseed	Wash-out	Placebo	
HDL (mg/dL)	46.5 (32.0–68.0)	44.0 (31.0–94.0)	54.4 (31.0–79.0)	43.5 (38.0–77.0)	*p* = 0.314
TG (mg/dL)	153.5 (61.0–413.0)	118.0 (87.0–352.0)	137.5 (89.0–457.0)	123.5 (61.0–244.0)	*p* = 0.911
ApoA1 (g/L)	1.35 (1.16–1.86)	1.49 (1.17–1.91)	1.77 (1.23–1.90)	1.97 (1.24–2.01)	*p* = 0.911
ApoB (g/L)	0.99 (0.54–1.94)	1.04 (0.55–1.42)	0.87 (0.56–1.62)	1.2 (0.58–1.69)	*p* = 0.117
ApoB/ApoA1	0.65 (0.42–1.49)	0.68 (0.28–0.99)	0.68 (0.42–1.14)	0.64 (0.41–1.27)	*p* = 0.771

Abbreviations: HDL, cholesterol high density; TG, triglycerides; ApoA1, apolipoprotein A1; ApoB, apolipoprotein B.

## Supplementary Materials

The following are available online at https://www.mdpi.com/2072-6643/12/4/1137/s1, Table S1: Detailed characteristics of study group, Table S2: Lipoprotein apheresis—detailed lipid parameters before and after sessions with relative reductions, Table S3: Changes in lipids parameters throughout the study.
