# Supplementary material for: Flaxseed (Linum Usitatissimum L.) Supplementation in Patients Undergoing Lipoprotein Apheresis for Severe Hyperlipidemia—A Pilot Study
Source: Nutrients. 2020 Apr 18;12(4):1137. doi: 10.3390/nu12041137 (PMC7231079; doi:10.3390/nu12041137)
Supplement: Supplementary file 1 [file nutrients-12-01137-s001.zip › nutrients-768428-supplementary.docx]

Table S1. Detailed characteristics of study group.

Conversion factors to SI units are as follows: cholesterol 0.02586 and triglycerides 0.0114. TC, LDL-C , HDL-C , TG are the highest values before LA initiation. Apo A1 — apolipoprotein A1; APOB — apolipoprotein B gene; BMI – body mass index; CAD — coronary artery disease; FH — familial hypercholesterolemia; GFR — glomerular filtration rate; HDL-C— high density lipoprotein cholesterol; IFG — impaired glucose tolerance; LA- lipoprotein apheresis; LDL-C — low density lipoprotein cholesterol; LLT- lipid lowering treatment; Lp(a) — lipoprotein (a); LVEF — left ventricular ejection fraction; NA — not applicable; ND – not done; R- rosuvastatin; TC — total cholesterol; TG — triglicerydes.

| *BASELINE HISTORY* | Patient 1 | Patient 2 | Patient 3 | Patient 4 | Patient 5 | Patient 6 |
| --- | --- | --- | --- | --- | --- | --- |
| *sex* | male | male | male | male | female | female |
| *Indication to LA* | isolated hyperlipoproteinemia (a) | isolated hyperlipoproteinemia (a) | FH  + hyperlipoproteinemia (a) | FH  +statin intolerance | FH + hyperlipoproteinemia (a) | FH  +statin intolerance |
| *FH-causing gene mutation* | NA | NA | APOB  (DLCN >8 pt) | ND  (DLCN >8 pt) | ND  (DLCN >8 pt) | ND  (DLCN >8 pt) |
| Cardiovascular disease | | | | | | |
| *CAD* | 1 | 1 | 1 | 1 | 1 | 0 |
| *CAD, age* | 46 | 46 | 61 | 49 | 64 | NA |
| *Stroke* | 0 | 0 | 0 | 0 | 0 | 0 |
| *Stroke, age of first* | NA | NA | NA | NA | NA | NA |
| *Carotid artery disease* | 0 | 0 | 0 | 0 | 0 | 0 |
| *Peripheral artery disease* | 0 | 0 | 0 | 0 | 0 | 0 |
| Cardiovascular risk factors | | | | | | |
| *Body weight, kg* | 70 | 91 | 80 | 110 | 68 | 53 |
| *BMI* | 22 | 30 | 29 | 32 | 31 | 23 |
| *Diabetes/IFG* | 0 | 0 | 0 | 0 | 0 | 0 |
| *Hypertension* | 0 | 0 | 1 | 1 | 1 | 0 |
| *Smoking history* | 1 | 0 | 0 | 1 | 1 | 0 |
| *Family history of premature CAD (in first-degree relative)* | 1 | 1 | 1 | 1 | 1 | 1 |
| *Chronic kidney disease* | 0 | 0 | 0 | 0 | 0 | 0 |
| *Lipid parameters and imaging results* | | | | | | |
| *TC (mg/dl)* | 245 | 216 | 191 | \| 234 \| \| --- \| | 252 | 367 |
| *LDL-C (mg/dl)* | 118 | 149 | 127 | 156 | 170 | 277 |
| *HDL-C (mg/dl)* | 77 | 49 | 49 | 39 | 56 | 48 |
| *TG (mg/dl)* | 244 | 317 | 187 | 735 | 272 | 408 |
| *Lp(a) (g/l)* | 3.15 | 1.99 | 2.36 | 0.06 | 0.15 | 0.15 |
| *Apo A1 (g/l)* | 1.61 | 1.76 | 1.59 | 1.31 | 1.51 | 1.66 |
| *Apo B (g/l)* | 0.73 | 0.79 | 1 | 1.28 | 1.33 | 1.97 |
| *LVEF (%)* | 35 | 50 | 65 | 40 | 60 | 65 |
| *Lipid-lowering treatment* | | | | | | |
| *Statin - age of initiation* | 46 | 46 | 35 | 41 | 38 | 31 |
| *Statin* | R 40 mg | R40 mg | R 40mg | 0 | R20 mg | 0 |
| *Ezetimibe* | 1 | 1 | 1 | 1 | 1 | 0 |
| *LA treatment, age of initiation* | 46 | 46 | 63 | 56 | 64 | 51 |
| *LA treatment, months* | 19 | 29 | 27 | 20 | 26 | 38 |

Table S2. Detailed lipid parameters before and after LA sessions and acute reductions (%).

Data are presented as  median (interquartile range). Abbreviations — see Supp Table 1.

| **Parameter** | **Patient 1** | **Patient 2** | **Patient 3** | **Patient 4** | **Patient 5** | **Patient 6** |
| --- | --- | --- | --- | --- | --- | --- |
| **LDL-C pre-apheresis, mg/dl** | 93  (80-101) | 59  (47-73) | 104  (100-111) | 125  (110-139) | 124  (114-136) | 214  (198-227) |
| **LDL-C post-apheresis, mg/dl** | 24  (21-28) | 16  (12-21) | 31  (30-33) | 44  (40-47) | 25  (23-31) | 66  (60-74) |
| **LDL-C, % reduction** | 71  (68-73) | 75  (73-77) | 71  (69-71) | 64  (62-67) | 79  (77-81) | 66  (64-71) |
| **TC pre-apheresis, mg/dl** | 183  (157-199) | 118  (11-141) | 162  (155-168) | 207  (197-222) | 211  (192-225) | 291  (278-303) |
| **TC post-apheresis, mg/dl** | 93  (80-95) | 56  (49-62) | 74  (71-79) | 88  (83-92) | 78  (70-84) | 113  (105-120) |
| **TC, % reduction** | 50  (46-52) | 55  (53-59) | 54  (52-56) | 58  (56-60) | 62  (61-66) | 62  (57-64) |
| **HDL-C pre-apheresis, mg/dl** | 61  (59-67) | 40  (37-42) | 43  (40-43) | 32  (30-34) | 55  (50-60) | 41  (38-43) |
| **HDL-C post-apheresis, mg/dl** | 52  (47-56) | 31  (29-35) | 35  (33-38) | 26  (25-28) | 40  (36-44) | 31  (27-36) |
| **HDL-C reduction, %** | 18  (9-20) | 20  (16-25) | 18  (12-20) | 19  (17-22) | 27  (23-32) | 26  (18-32) |
| **TG pre-apheresis, mg/dl** | 170  (134-205) | 135  (110-177) | 80  (70-89) | 406  (304-517) | 144  (112-169) | 215  (181-253) |
| **TG post-apheresis, mg/dl** | 68  (54-79) | 44  (48-55) | 37  (35-44) | 126  (114-150) | 43  (40-46) | 85  (74-108) |
| **TG reduction %** | 55  (46-65) | 67  (64-69) | 54  (47-59) | 68  (60-74) | 70  (67-74) | 59  (52-65) |
| **Lp(a)  pre-apheresis, g/l** | 2.28  (1.98-2.50) | 1.75  (1.48-1.96) | 1.56  (1.46-1.86) | 0.05  (0.04-0.05) | 1.52  (1.32-1.78) | 0.11  (0.10-0.12) |
| **Lp(a)  post-apheresis, g/l** | 0.74  (0.66-0.98) | 0.42  (0.40-0.47) | 0.47  (0.41-0.55) | 0.02  (0.02-0.02) | 0.30  (0.28-0.33) | 0.04  (0.03-0.05) |
| **Lp(a), reduction %** | 64  (58-70) | 76  (73-77) | 72  (69-73) | 60  (50-64) | 81  (79-82) | 64  (58-67) |
